# Multimodal MRI radiomics based on habitat subregions of the tumor microenvironment for predicting risk stratification in glioblastoma

**DOI:** 10.1371/journal.pone.0326361

**Published:** 2025-06-27

**Authors:** Han Wang

**Affiliations:** Department of Ultrasound Medicine, Nanjing Drum Tower Hospital, Affiliated Hospital of Nanjing University Medical School, Nanjing, China; University of Maryland College Park, UNITED STATES OF AMERICA

## Abstract

**Objective:**

Accurate prediction of glioblastoma (GBM) progression is essential for improving therapeutic interventions and outcomes. This study aimed to develop and validate an integrated clinical-radiomics model to predict overall survival (OS) and evaluate the risk of disease progression in patients with isocitrate dehydrogenase-wildtype GBM (IDH-wildtype GBM).

**Materials and Methods:**

The data of 423 IDH-wildtype GBM patients were retrospectively analyzed. Radiomic features were extracted from preoperatively acquired MR images. Least absolute shrinkage and selection operator-Cox proportional hazards (LASSO-Cox) regression was used to identify radiomic features significantly associated with OS and calculate a risk score and construct a radiomic signature for each patient. Kaplan‒Meier survival analysis and the log-rank test were used to compare survival between the high-risk and low-risk groups. A clinical‒radiomic model and a nomogram were developed on the basis of the results of multivariable Cox proportional hazards regression and were evaluated with the concordance index (C-index).

**Results:**

Radiomics models were developed on the basis of feature extracted from the three sub-regions individually, and a multiregional radiomics model was established by aggregating 16 features selected from these subregions. Kaplan-Meier survival analysis indicated that the high-risk group exhibited significantly worse outcomes than the low-risk group did (*p* < 0.05). The C-index of the multiregional radiomics model was the highest. Univariable Cox regression analysis revealed that the risk score, age, and extent of gross total resection (GTR) were significant prognostic factors for OS in GBM patients. According to the C-index, the combined clinical‒radiomic model outperformed the standalone radiomic and clinical models. The multifactor nomogram showed high accuracy in predicting the OS rates of preclinical GBM patients at 3 months, 6 months, 1 year, and 3 years in both the training and test cohorts.

**Conclusions:**

The integrated model combining clinicopathological data with a radiomic signature achieves good risk stratification and survival prediction in GBM and thus could be an important tool in clinical practice.

## Introduction

Glioblastoma (GBM) is considered the most aggressive type of primary malignant brain tumor [1]. According to the 2021 World Health Organization (WHO) classification of central nervous system (CNS) tumors, isocitrate dehydrogenase (IDH)-wildtype GBM is designated CNS-WHO grade 4 [2]. The outcomes of individuals with IDH-wildtype GBM remains poor, as its 5-year survival rate is less than 7% [3,4]. The most important prognostic factors for survival in GBM patients include patient age, Karnofsky performance score, extent of gross total resection (GTR), and WHO tumor grade [5]. However, the application of these factors in clinical practice is limited due to their reliance on invasive procedures and specialized resources [6–8].

Radiomics, a technique that converts medical images into high-dimensional data, has become a powerful noninvasive approach in cancer diagnosis and prognosis prediction [9,10]. This technique allows the extraction of predictive imaging phenotypes from preoperative magnetic resonance imaging (MRI) that, in patients with GBM, are indicative of the biological behavior of the tumor and are strongly correlated with overall survival (OS) [11–13]. However, the integration of prognostically important imaging features with clinical and pathological data remains challenging [14,15].

Prior research has focused predominantly on extracting radiomic features from the entire tumor, and few studies have investigated the imaging characteristics of distinct spatial regions of GBM lesions. Moreover, diffusion tensor imaging (DTI) offers a greater understanding of tumor genetics than conventional structural MRI modalities such as contrast-enhanced T1-weighted imaging (T1WI-CE) and T2 fluid-attenuated inversion recovery (FLAIR). However, existing studies largely relied on structural MRI for their analyses. To address these limitations, we employed automated techniques to segment GBMs into three habitat subregions of the tumor microenvironment while integrating conventional MRI sequences with DTI fractional anisotropy (FA) parameters. This methodology was designed to develop a comprehensive radiogenomic model that can accurately stratify risk and be used to evaluate the outcomes of GBM patients, thereby improving personalized treatment strategies and survival outcomes.

## Methods and materials

### Patients

The dataset utilized in this study was acquired from The Cancer Imaging Archive (TCIA), specifically from the “University of Pennsylvania Glioblastoma Imaging, Genomics, and Radiomics” (UPenn-GBM) dataset [16]. It included preoperative dynamic contrast-enhanced (DCE)-MRI scans from 671 patients diagnosed with GBM through biopsy with comprehensive tumor data. The cohort was randomly divided into training and testing cohorts at a 7:3 ratio. The publicly available database was used in accordance with the citation guidelines and data usage policies outlined on the TCIA website. Institutional review board approval was waived for this study because of the inaccessibility of patient identifiers to database users.

The inclusion criteria for this study were as follows: (1) preoperative MRI data, including T1WI-CE, FLAIR, and DTI-FA parameter map, with complete sequences, clear images, and comprehensive clinical and pathological information; and (2) complete OS, clinical, and pathological information. The exclusion criteria were as follows: (1) poor image quality (e.g., presence of motion artifacts); (2) previous brain surgery; and (3) incomplete OS, clinical, or pathological data.

**Fig 1:**
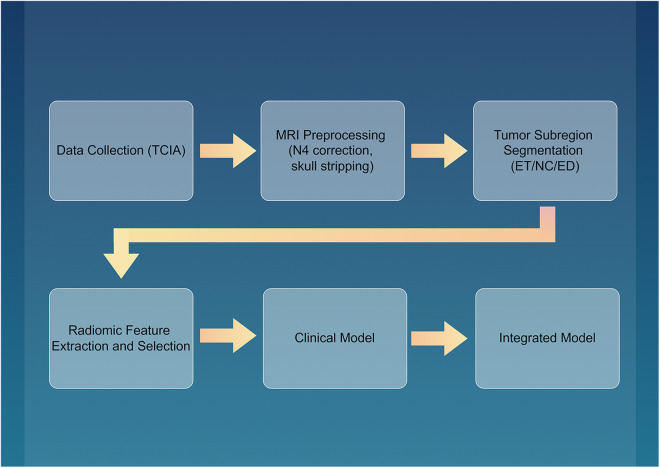
Study flowchart depicting the procedure.

After excluding patients without MRI or OS data, 423 patients with IDH-wildtype GBM were included. The study flowchart is shown in Fig 1.

### MR image acquisition and analysis

Preoperative MRI scan parameters were sourced from the TCIA, with MRI scanners operating at 1.5 T or 3.0 T. All patients were scanned with the T1WI-CE sequence (matrix size 192 × 256 × 192, voxel size 0.98 × 0.98 × 1.00, TR/TE 1760 ms/3.1 ms), FLAIR sequence (matrix size 192 × 256 × 60, voxel size 0.94 × 0.94 × 3.00, TR/TE 9420 ms/141 ms), and DTI sequence (matrix size 128 × 128 × 40, voxel size 1.72 × 1.72 × 3.00, TR/TE 3100–8000 ms/104 ms). The T1WI-CE images were obtained following an injection of gadobenate dimeglumine at a dose of 0.3 ml/kg for contrast enhancement. The FA map was generated from the DTI images with postprocessing software [17]. The scanner specifications and MRI acquisition parameters have been documented in previous studies [16,18,19].

A comprehensive preprocessing pipeline was established to standardize the data and ensure consistency in the radiomic feature extraction. This pipeline includes the following steps: image reorientation, resampling to an isotropic resolution of 1 mm3, intensity normalization using the N4 bias field correction algorithm, skull stripping, and defacing. The tumor subregions were delineated using a combination of deep learning algorithms and label fusion techniques followed by manual refinement by expert radiologists. These procedures followed established radiomic research methodologies, such as those used in the UPenn-GBM dataset, ensuring that our findings are both robust and reproducible despite potential differences in the acquisition parameters within the study dataset.

### Lesion segmentation and radiomics feature extraction

A fully automated approach involving label fusion from multiple deep learning algorithms was used to segment distinct tumor subregions histologically. The segmentation focused primarily on delineating the enhanced (ET), necrotic core (NC), and edema (ED) regions. Radiographically, the ET and NC regions were identified on T1WI-CE as areas of hyperintense and hypointense signals, respectively, with respect to the signals on T1-weighted images and in the normal white matter. The NC region includes the unenhanced or faintly enhanced tumor core, such as transitional/prenecrotic and necrotic areas, which are typically surgically resected along with the ET. The ED region is characterized by an abnormal, hyperintense signal observed on FLAIR imaging. All the segmentation results were manually reviewed and corrected using ITK-SNAP to ensure accuracy [20,21]. Computer-extracted imaging features were obtained from the TCIA. These data included commonly published features from the literature as well as uniquely identified features.

Following segmentation, quantitative imaging phenomic (QIP) features were derived from each tumor subregion with the Cancer Imaging Phenomics Toolkit (CaPTk) [22–24] in accordance with the guidelines established by the Image Biomarker Standardisation Initiative (IBSI) [25]. A total of 145 features were extracted for each subregion, encompassing five categories:

(1) Intensity-based features, or first-order statistics (e.g., mean, median, maximum, minimum, standard deviation, skewness, kurtosis).
(2) Histogram-related features, which describe the range and distribution of gray-level intensities.
(3) Volumetric measurements, including shape such as elongation, perimeter, principal component axes, and area/volume for 2D/3D data.
(4) Morphological parameters, reflecting the geometric properties of the tumor.
(5) Textural descriptors, including indices derived from the gray-level co-occurrence matrix (GLCM), gray-level run-length matrix (GLRLM), gray-level size zone matrix (GLSZM), neighborhood gray-tone difference matrix (NGTDM), and local binary pattern (LBP).

### Establishment of clinical model

Univariable and multivariable Cox regression analyses were conducted to identify clinical predictors of survival in GBM patients. Variables with a p value < 0.05 in the univariable analysis were included in the multivariable model. Finally, variables with a p value < 0.05 in the multivariable analysis and a variance inflation factor (VIF) less than 5 were selected to develop the clinical predictive model.

### Establishment of the radiomic model

In the training cohort, dimensionality reduction and feature selection were performed through a series of steps. First, all feature vectors were standardized with z score normalization to allow uniform comparisons across the features. Then, the Pearson correlation coefficient was calculated to assess interfeature correlations. For two features with a correlation coefficient greater than 0.9, one was randomly excluded from further analysis, thereby addressing potential multicollinearity issues. Univariable Cox regression analysis was subsequently conducted on the retained features; those demonstrating a p value of less than 0.05 in this analysis were further evaluated with least absolute shrinkage and selection operator-Cox proportional hazards (LASSO-Cox) regression and 10-fold cross-validation to identify the radiomic features most significantly associated with OS. The selected features were then linearly combined after weighting with their coefficients to compute the radiomics score (rad-score) for each subject. The abovementioned steps were separately performed for the ED, ET, and NC regions to develop individual subregion radiomic models. The features extracted from these three regions were subsequently integrated to construct a multiregional radiomics model.

### Establishment and evaluation of the integrated model

Three models were developed using both radiomic and clinical features. Harrell’s concordance index (C-index) was used to evaluate model performance in both the training and testing groups. The patients were then categorized into high-risk and low-risk groups on the basis of the median value obtained from the integrated model. Kaplan‒Meier survival analysis and the log-rank test were used to assess differences in the time to progression (TTP) between the two groups. A multifactor predictive nomogram incorporating both clinical and radiomic features was developed from the results of LASSO regression and multivariable Cox proportional hazards analysis. The performance of the nomogram was evaluated with decision curve analysis (DCA) and receiver operating characteristic (ROC) curve analysis.

### Statistical analysis

Statistical analysis was performed using SPSS Version 25.0, Python 3.5.6, and R 3.5.3 software. Continuous variables were compared between groups with the independent samples t test, while categorical variables were compared between groups with the chi-square test or Fisher’s exact test. A two-tailed p value < 0.05 was considered to indicate statistical significance.

## Results

### Subjects characteristics

In total, 423 patients with IDH-wildtype GBM were included. The clinical data were shown in Table 1.

**Table 1:** Comparison of clinical data in training set and validation set.

Patient characteristics	Training cohort (n = 298)	Testing cohort (n = 125)	*p*-value
Age	63.48 ± 11.50	63.46 ± 11.07	0.941
Gender			0.623
male	181 (60.74)	72 (57.60)	
female	117 (39.26)	53 (42.40)	
MGMT			0.364
Unmethylated	100 (33.56)	51 (40.80)	
Indeterminate and methylated	60 (20.13)	22 (17.60)	
Not available	138 (46.31)	52 (41.60)	
GTR			0.889
Less than 90%	108 (36.24)	47 (37.60)	
More than 90%	178 (59.73)	72 (57.60)	
Not available	12 (4.03)	6 (4.80)	

Abbreviation: A cohort of 423 patients diagnosed with isocitrate dehydrogenase-wildtype glioblastoma (IDH-wildtype GBM) was randomly allocated into the training set and testing set. Subsequently, a comparative analysis of the clinical data from these two sets was conducted. MGMT: 6-methylguanine-DNA methyltransferase; GTR: Gross total resection.

### Clinical model construction

Predictive risk factors identified through univariate Cox regression analysis were included in multivariable Cox regression analysis, and subject to collinearity evaluation. A clinical model was constructed using the independent factors identified from multivariable Cox regression analysis, with criteria of *p* < 0.05 and a VIF less than 5. The results showed that age (HR: 1.023, 95% CI: 1.012–1.034, *p* < 0.05; VIF < 5) and GTR over 90% (HR: 0.702, 95% CI: 0.570–0.864, *p* < 0.05; VIF < 5) were independent predictors of OS. Therefore, the clinical model was based on these independent prognostic factors.

### Radiomics model construction

**Fig 2:**
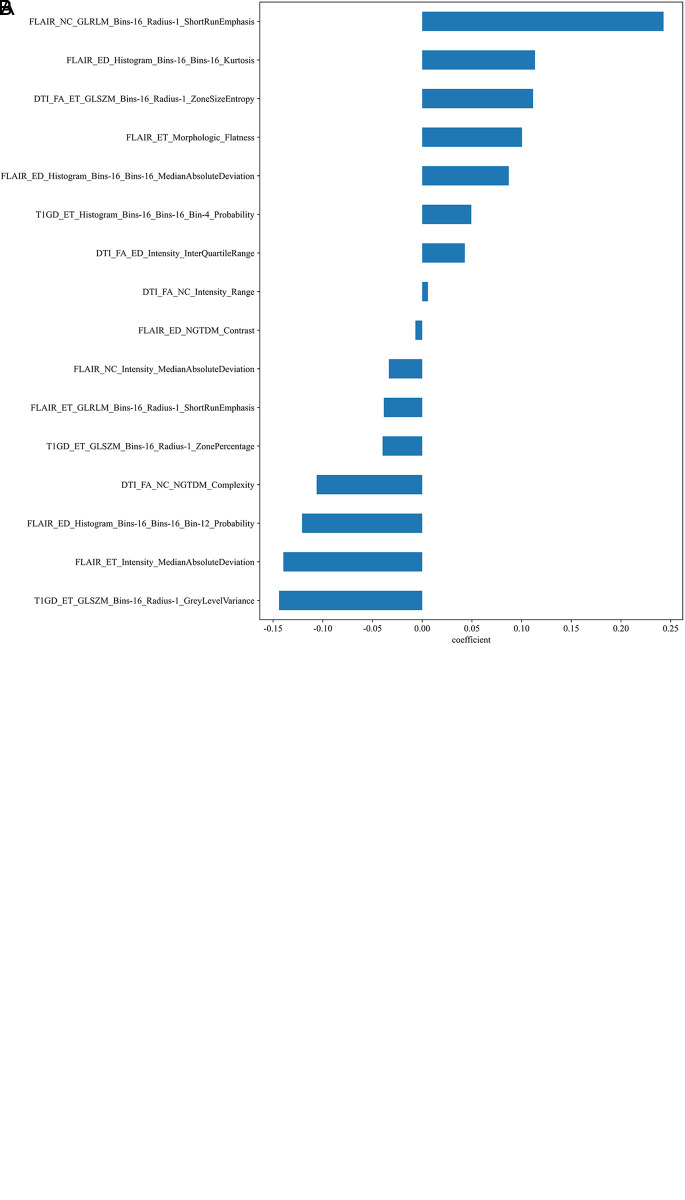
Feature selection and coefficient analysis in LASSO-Cox regression model for GBM survival prediction. (a) Change in the coefficients of variables in the LASSO model with varying values of the regularization parameter (λ). The optimal λ was selected using 10-fold cross-validation, as indicated by the vertical dashed line. (b) Coefficients of the 16 features selected by LASSO Cox regression. These features were retained based on their contribution to the prediction of overall survival in GBM patients.

Separate sets of features were extracted and dimensionally reduced from the three habitat subregions. Finally, 16 radiomics features with the strongest association with OS were selected, including 7 features from the ET, 5 from the ED, and 4 from the NC. The rad-score for each subject was computed as the linear combination of these selected features, each weighted by its coefficient (Fig 2).

Separate radiomic models were developed for the three habitat subregions as well as the multiregional radiomics model. Kaplan–Meier survival analysis indicated that the high-risk group exhibited significantly worse survival than the low-risk group did (P < 0.05). The C-index for the multiregional radiomic model was higher than that of each of the habitat radiomic models, with a value of 0.668 in the training set and 0.626 in the test set. The next best model was that based on the ET region, which had a C-index of 0.622 in the training set and 0.602 in the test set. The ED region model demonstrated the lowest performance, with a C-index of 0.605 in the training set and 0.574 in the test set (Table 2).

**Table 2:** Evaluation of radiomics, clinical, and clinical-radiomics model.

Models	Training cort	Testing cort
	C-index	C-index
Radiomics (ET)	0.622 (0.567–0.677)	0.602 (0.516–0.688)
Radiomics (ED)	0.605 (0.549–0.660)	0.574 (0.487–0.660)
Radiomics (NC)	0.621 (0.566–0.677)	0.572 (0.486–0.659)
Multiregional radiomics^*^	0.668 (0.614–0.721)	0.626 (0.541–0.711)
Clinical^*^	0.632 (0.577–0.687)	0.589 (0.503–0.675)
Clinical-radiomics combined	0.690 (0.637–0.742)	0.637 (0.553–0.722)

Abbreviation: The Kaplan-Meier survival analysis based on the three sub-regions radiomics, the entire tumor radiomics, clinical and clinical-radiomics models were used to compare survival differences between high-risk and low-risk groups and were evaluated by the concordance index (C-index). ET: enhancement: ED: edema: NC: necrotic core.

* Represents Clinical-radiomics combined model were constructed using multiregional radiomics and clinical model.

### Establishment and evaluation of the combined model

**Fig 3:**
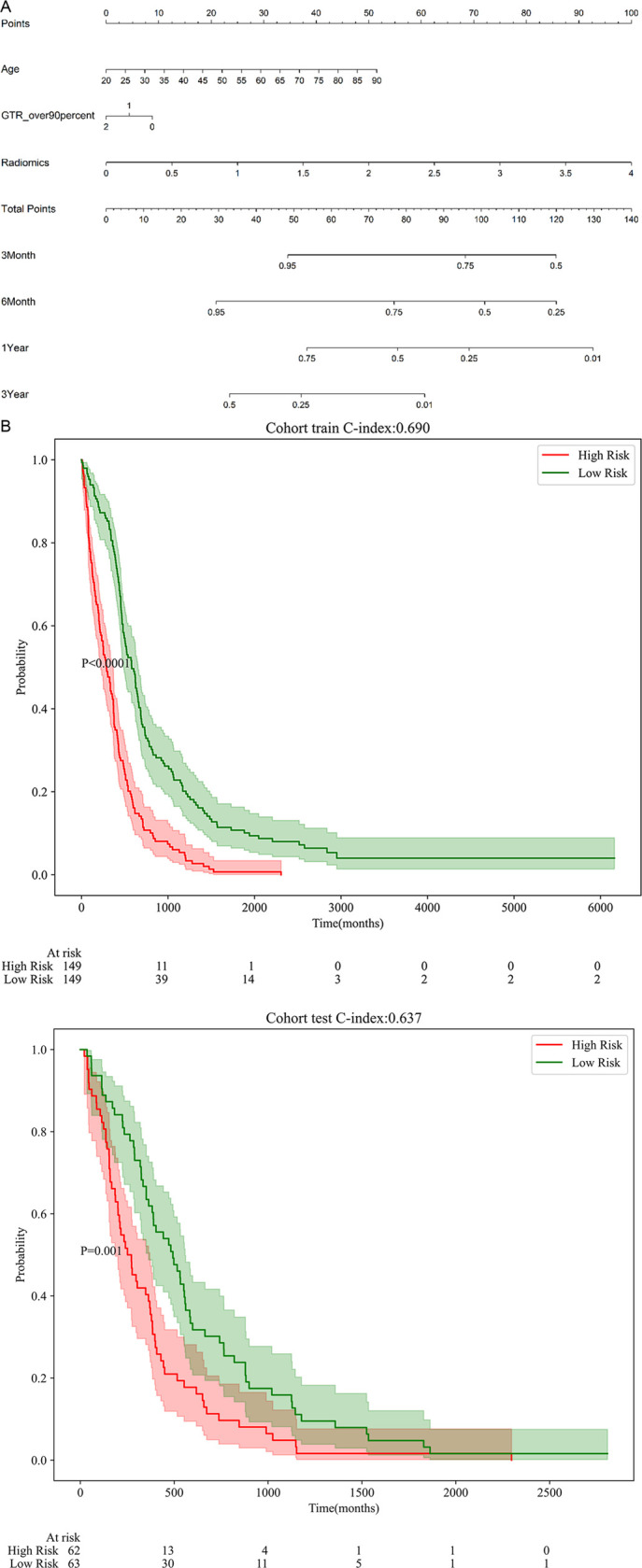
Development and validation of a multipredictor nomogram for GBM survival prediction. (a) Multi-predictor nomogram for evaluating overall survival (OS) in GBM patients. The nomogram integrates selected radiomic features and clinical variables, allowing for individualized survival predictions. (b) Kaplan-Meier survival curves in the training cohort, stratified by risk groups derived from the nomogram. The log-rank test was used to assess statistical significance between groups. (c) Kaplan-Meier survival curves in the test cohort.

The C-index was used to assess the performance of the clinical model, the whole-tumor radiomic model, and the integrated clinical-radiomics models in both the training and testing cohorts. The integrated clinical-radiomics model, which demonstrated the best performance (Table 2), was developed by integrating the rad-score from the radiomic model with the two clinical features used to construct the clinical model and was used to predict the 3-month to 3-year OS rates. Furthermore, a nomogram was constructed to facilitate use of the model in an easily visualized form (Fig 3).

### Validation of a multifactor predictive nomogram

**Fig 4:**
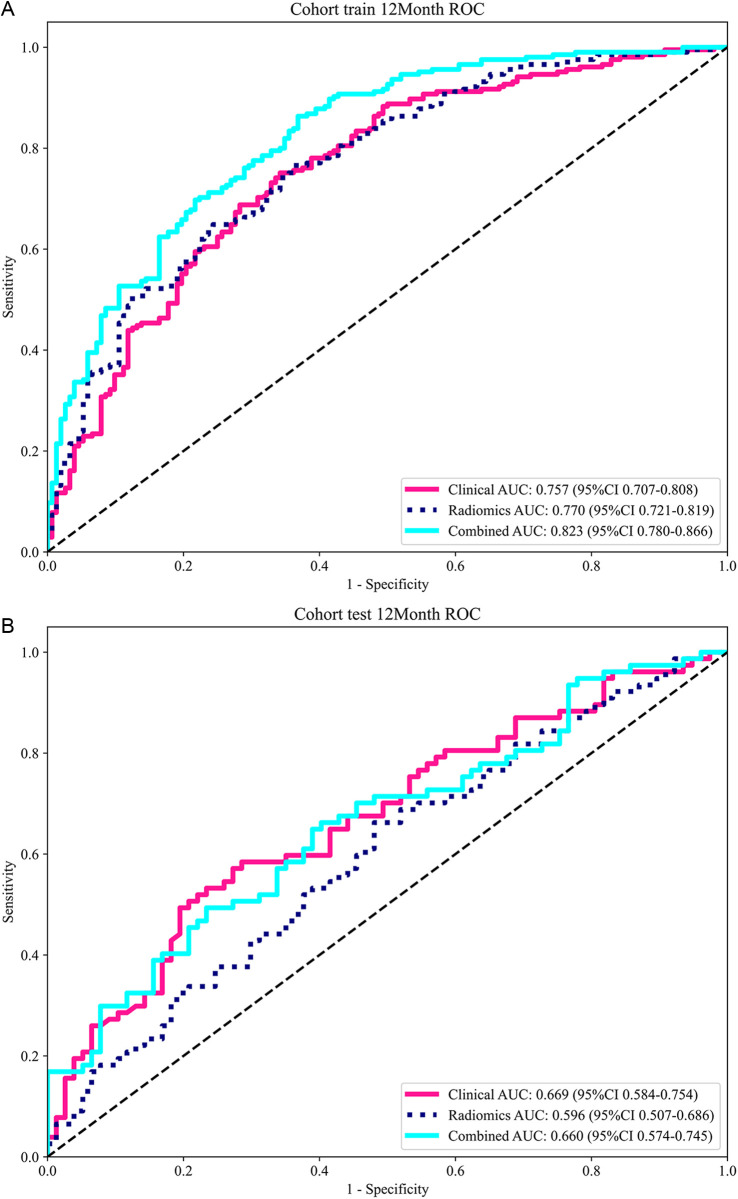
ROC analysis of 1-year survival prediction accuracy for the integrated clinical-radiomics model. Receiver operating characteristic (ROC) curve for the model’s performance in predicting 1-year survival. The area under the curve (AUC) is provided as a measure of predictive accuracy: (a) training set, (b) testing set.

**Fig 5:**
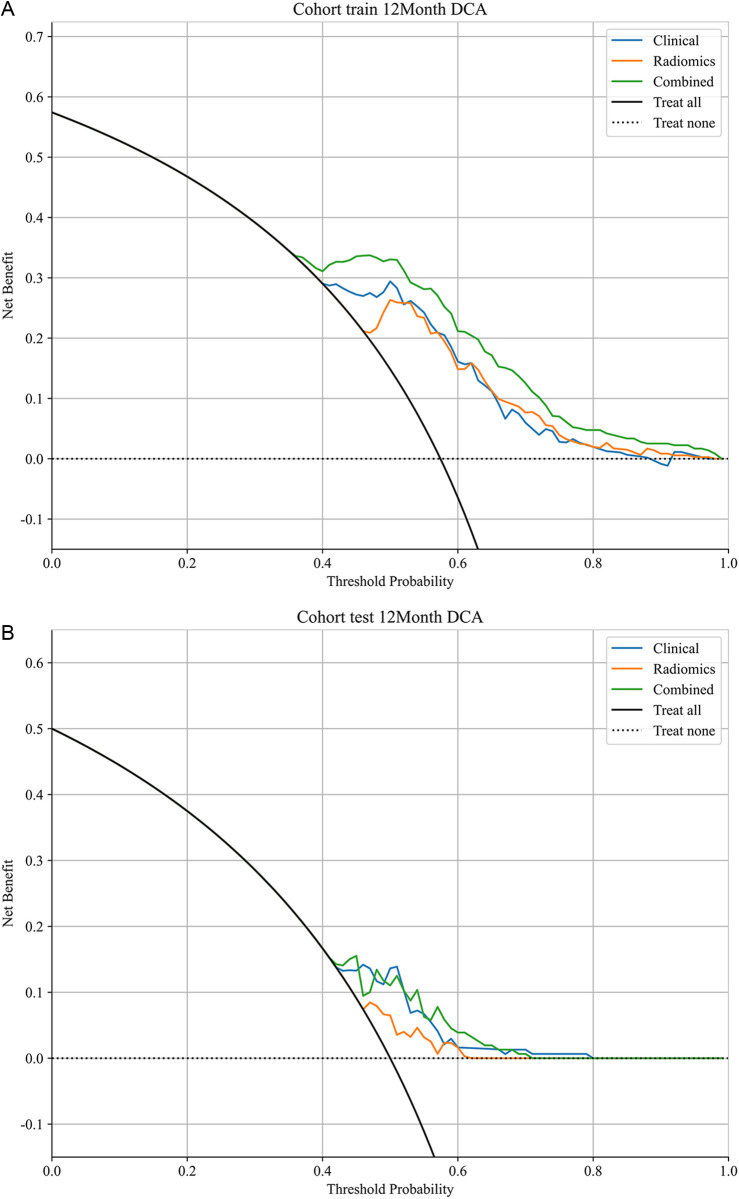
Decision curve analysis (DCA) evaluating the clinical utility of the integrated model for 1-year survival prediction. Decision curve analysis (DCA) for the model’s clinical utility in predicting 1-year survival. The net benefit at various threshold probabilities is shown, compared to default strategies (treat all, treat none): (a) training set, (b) testing set.

**Fig 6:**
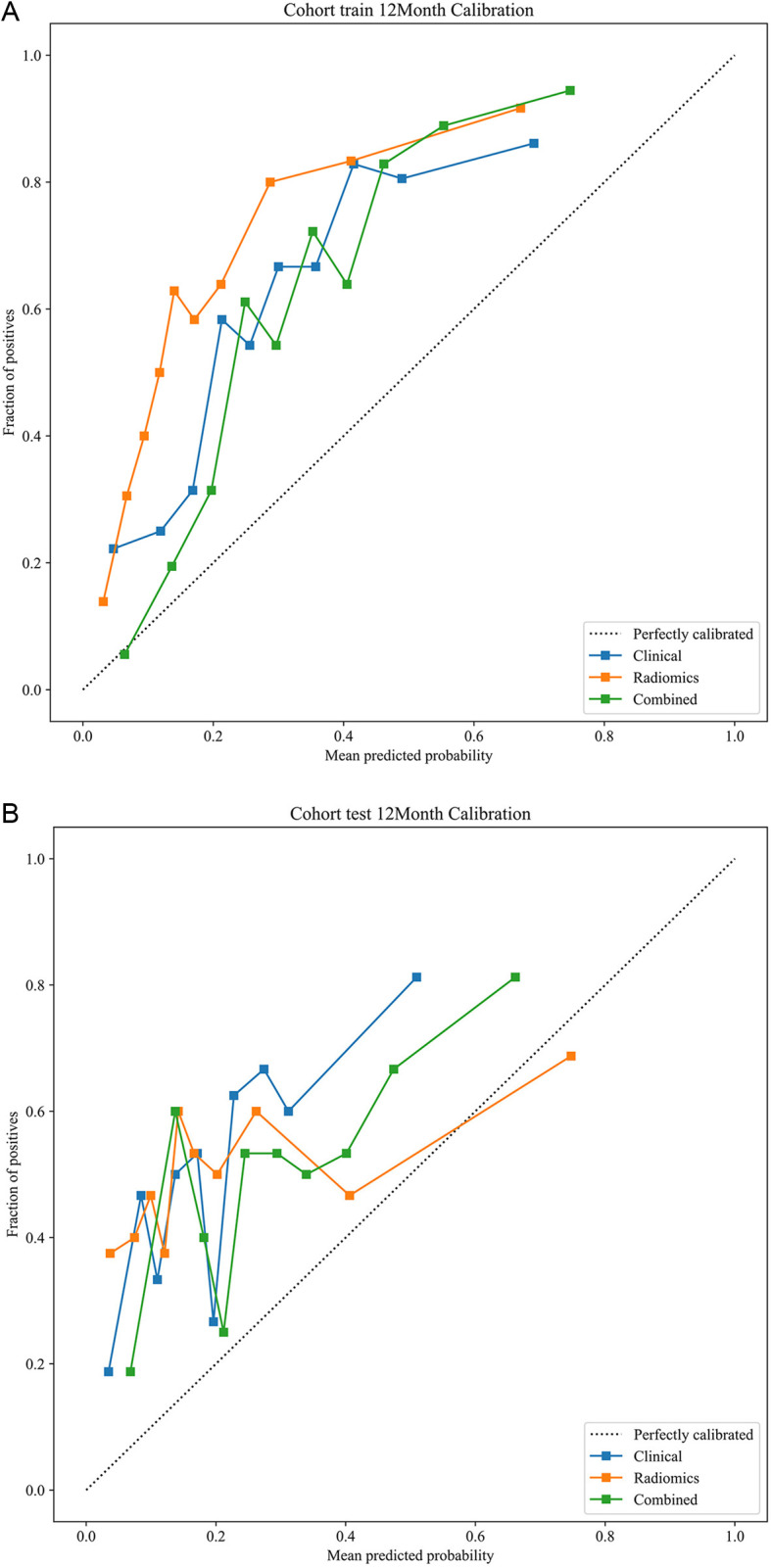
Calibration analysis of the integrated model for overall survival prediction. Overall survival calibration curves in the training cohort and testing cohort. The dashed lines are ideal calibration curves, and the solid lines are extracted calibration curves derived by calibration process. (a) training set, (b) testing set.

ROC curve analysis (Fig 4, Table 3) indicated that the multifactor predictive nomogram performed very well for the OS rate of preclinical GBM patients at 3 months, 6 months, 1 year, and 3 years within both the training and testing cohorts. The predictive performance was most robust for the 1-year OS rate, with AUC values of 0.789 and 0.668 in the respective cohorts. Furthermore, DCA (Fig 5) revealed that the multifactor nomogram provided the greatest net clinical benefits in predicting the 1-year OS rate in both cohorts. Calibration curves were used to assess the calibration ability of the nomogram (Fig 6).

**Table 3:** AUC of the model for predicting the probability of survival of 3 months, 6 months, 1 year, and 3 years.

Models		Training cort		Testing cort	
		AUC(95% CI)	Accuracy	AUC (95% CI)	Accuracy
3-month	Clinical	0.746 (0.66–0.83)	0.554	0.595 (0.431–0.758)	0.632
	Radiomics	0.737 (0.654–0.819)	0.782	0.604 (0.421–0.785)	0.64
	Combined	0.788 (0.708–0.867)	0.832	0.613 (0.437–0.788)	0.776
6-month	Clinical	0.755 (0.686–0.823)	0.685	0.623 (0.506–0.738)	0.664
	Radiomics	0.738 (0.670–0.804)	0.732	0.655 (0.537–0.773)	0.68
	Combined	0.789 (0.725–0.853)	0.782	0.688 (0.579–0.797)	0.592
1-year	Clinical	0.717 (0.657–0.777)	0.678	0.637 (0.539–0.7342)	0.616
	Radiomics	0.747 (0.690–0.803)	0.654	0.647 (0.551–0.743)	0.624
	Combined	0.789 (0.735–0.841)	0.752	0.668 (0.573–0.761)	0.616
3-year	Clinical	0.62 (0.531–0.707)	0.671	0.701 (0.554–0.848)	0.608
	Radiomics	0.767 (0.681–0.853)	0.815	0.706 (0.529–0.882)	0.752
	Combined	0.744 (0.665–0.821)	0.577	0.762 (0.594–0.928)	0.904

By integrating the radiomics based the entire tumor with the two clinical features derived from the clinical model, the combined model facilitated the prediction of overal survival (OS) probabilities over a 3-month to 3-year period.

## Discussion

### MRI sequence selection and habitat imaging

In this study, we systematically for the first time integrated conventional and functional MRI sequences, specifically T1WI-CE and FLAIR, with DTI-FA parameters to extract radiomic features from distinct tumor ecological subregions, namely ET, NC, and ED [26–28]. This multi-sequence model captures heterogeneous pathophysiological information, overcoming the noise sensitivity and biological oversimplification inherent in single-modality approaches such as Yan et al.’s DTI-only model [29] and Viet Huan Le et al.’s conventional MRI squences (T1WI, T1WI-CE, T2WI, FLAIR) [30].

In terms of ROI segmentation region selection, the automated segmentation in our study contrasts with prior work by Ruchika Verma et al., which relied on manual or semi-automated techniques [31]. This approach enhances reproducibility and minimizes human bias, ensuring more consistent and reliable results. The research conducted by Le NQK further illustrated the progression of radiomics through the incorporation of deep learning techniques, facilitating automated segmentation, feature extraction, and multi-feature fusion to improve predictive accuracy [32]. Achieving these advancements incrementally constitutes our forthcoming research objective.

Habitat imaging represents an innovative approach to tumor characterization, involving the segmentation of tumors into distinct subregions [33,34]. Each subregion exhibits unique biological behaviors driven by growth, proliferation, and treatment resistance [35]. In this study, we developed radiomics models for GBM risk stratification, focusing on both subregional and global tumor areas. Among the three subregions, the ET region was the most significant contributor. This prominence is likely due to the ET region’s representation of active tumor proliferation and angiogenesis, which are typically linked to aggressive disease behavior [36,37]. Nonetheless, the radiomics model encompassing the entire tumor region incorporated more comprehensive data, resulting in superior predictive performance [38].

### Risk segmentation based on model construction methods

The patients were stratified on the basis of their risk scores, and Kaplan‒Meier survival analysis and the log-rank test were used to compare survival between the high- and low-risk groups in both the training and test cohorts. Kaplan–Meier analysis yielded a p value < 0.05, indicating that the OS rates of the low-risk group were significantly greater than those of the high-risk group. Among the features extracted and subjected to dimensionality reduction, the ShortRunEmphasis (SRE) derived from the NC region; the ZoneSizeEntropy (ZSE) derived from the ET region; and the Kurtosis obtained from the ED region were the most significant contributors. Both the SRE and ZSE are GLRLM features; a high SRE value indicates high heterogeneity, which is associated with high invasiveness and a hypoxic microenvironment [39,40], while a high ZSE value suggests a complex tumor microenvironment[ [41]. Kurtosis, which describes the “sharpness” of the image pixel intensity distribution, may indicate cell-dense or metabolically active areas when the value is high and is associated with a tendency of the tumor to proliferate and metastasize [42].

With respect to model development, various studies have explored the application of random forest and other machine learning classifiers to predict patient survival [43,44]. Survival analysis is often treated as a binary classification task, but this approach overlooks the importance of time. Binary classification predicts outcomes at a specific time point, whereas real-world survival analyses involve continuous survival times influenced by factors such as age, sex, and disease status; ignoring these factors can reduce the model’s accuracy. In this study, we employed Cox regression to construct an OS model, considering risk variables as continuous variables, and developed a model that is not dependent on any threshold values.

### Clinical value and application of the radiomic model

The radiomic model proposed in this study offers significant potential for integration into clinical workflows for GBM treatment. For example, the developed nomogram could function as a decision-support tool, allowing clinicians to stratify patients into distinct risk groups for developing more personalized treatment plans. High-risk patients identified by the model, for example, could be prioritized for aggressive therapeutic interventions, such as combination therapies, or enrollment in clinical trials targeting treatment-resistant GBM. Conversely, low-risk patients could undergo more conservative monitoring, thereby avoiding exposure to potentially toxic treatments. Moreover, the model’s ability to predict 1-year survival rates may aid in the identification of patients likely to benefit from palliative care, thus improving quality of life and optimizing resource allocation. Future studies should consider developing user-friendly software interfaces to enable seamless integration of our proposed model into routine clinical practice.

### Limitations and future directions

This study has certain limitations. First, our study included 423 patients. While datasets of this size have been effectively employed in previous research [45,46], increasing the size of the cohort and including individuals from diverse populations would improve the robustness and clinical applicability of our model. Furthermore, although our internal validation process involved a careful division of the patients into training and test cohorts—a conventional method in radiomics research when external datasets are unavailable—external validation using independent datasets is crucial for assessing the generalizability of our model [47]. Moreover, our retrospective analysis did not specifically address the impact of missing or incomplete data, an important issue in retrospective studies. This limitation has the potential to introduce selection bias and restrict the statistical power of the model. In future research, we intend to collaborate with multiple institutions to generate a more comprehensive dataset, employ robust data imputation techniques to address missing values and improve data completeness, and conduct external validation with the data from independent cohorts to determine the generalizability of the models.

In addition, our reliance on LASSO-Cox regression for feature selection may not fully capture any nonlinear relationships between features and outcomes. However, LASSO-Cox regression was selected due to its ability to handle high-dimensional data, produce clinically interpretable results, and reduce overfitting, as reported by previous radiomic studies [48,49]. Future work will explore nonlinear approaches to further improve the robustness and predictive power of our models.

Finally, numerous studies have shown that the integration of imaging genomics with clinical, pathological, and genetic data can markedly improve model performance [50,51]. Future research should investigate the potential of this multimodal integration among radiomic, genomic and transcriptomic data.

## Conclusion

A GBM survival prediction model was developed by integrating clinical features with MRI radiomic features, resulting in good predictive performance. This model can improves clinicians’ ability to assess risk in GBM patients accurately, thereby facilitating personalized treatment planning.

### Institutional review board statement

The dataset used in this study was obtained from The Cancer Imaging Archive (TCIA). The use of the public database complied with the citation requirements and data use policies listed on the public portal of the TCGA-TCIA website. The study was exempt from institutional review board review and approval because patient identifiers were not available to database users.
